# Responsibility as an Ethics and Sustainability Element during the Pandemic

**DOI:** 10.3390/bs13070615

**Published:** 2023-07-24

**Authors:** Eugenia Țigan, Monica Lungu, Oana Brînzan, Radu Lucian Blaga, Ioana Anda Milin, Simona Gavrilaș

**Affiliations:** 1 Faculty of Food Engineering, Tourism and Environmental Protection, “Aurel Vlaicu” University of Arad, 2-4 Elena Drăgoi Str., 310330 Arad, Romania; eugenia.tigan@uav.ro (E.Ț.); monica.lungu@uav.ro (M.L.); oana.brinzan@uav.ro (O.B.); 2 Faculty of Economics, “Aurel Vlaicu” University of Arad, 77 Revoluției Bdl., 310032 Arad, Romania; 3 Faculty of Management and Rural Tourism, University of Life Sciences “King Mihai I” from Timisoara, 300645 Timisoara, Romania

**Keywords:** change, COVID-19, crisis, deonthology, liability

## Abstract

This article addresses two perspectives of responsibility: as an element of taking action and as ethics towards one’s fellow citizens and the environment. These aspects have been used since the beginning of the pandemic. In this context, we wanted to determine the mechanism that triggers increased responsibility. We have considered two possible initial processes: one external and one learned from the family in early childhood or trained/learned during studies. Based on that, three concerns were brought to our attention. First, we targeted the impact of the pandemic on respondents’ degree of responsibility for their livelihoods. Second, we wanted to determine to what extent the new situation increased the interviewee’s involvement only in some activities. The third statement called into question the causal relation between the influence of extrinsic elements on a highly educated person’s degree of responsibility. The focus group was the active population of the industrial sector in the North–West border area of Romania. The data obtained are the result of the sociological survey implementation. The people in the region were subject to several new limiting external factors. The results show that the unique challenges up to that point made them more accountable for their actions in a situation that affected them directly (pandemic). It also can be underlined that lifelong education is important in forming healthy principles of responsibility.

## 1. Introduction

We live in a world that is dealing with multiple global crises: a health crisis, an economic crisis, and a climate crisis. Pandemic situations are consequences of population growth and uncontrolled urbanization, resource overexploitation that leads to environmental degradation, climate change, and adding vectors like international travel. It is important now to understand what we have done right and what we have done wrong.

Even after so many years of a sustainable development policy being in practice, the major concern is that present designed goals do not include an explicit ethical dimension. It is more implicit with missing expected moral behaviors, follow-up principles, values, and ethical standards that must be pursued. So, which are the key elements that will lead to responsible behavior, and understanding how responsible behavior is formed is a stringent necessity.

The main theme represented the responsibility shown by the population of Romania’s North–West border area. The research was carried out in three instances over the last two years.

Thus, for the elaboration of the work, we developed a societal survey for the segment of inhabitants residing in the functional industrial region of North–West Romania. The territory is located at the intersection of Europe’s North–South and East–West axes [1]. The sector is in the border area, surrounded by the counties of Arad, Bihor, Satu Mare, and Maramureș [2] and by the borders of Hungary and Ukraine.

The facts have shown that crises, such as COVID-19 [3,4] and military conflicts, mainly affect the people and the environment of the border surroundings [5,6,7]. The population in this sector is the most vulnerable to adverse situations, having to take the first measures of restriction and protection, both in the case of the pandemic and the case of the Russia–Ukraine conflict. Furthermore, they had to face a major wave of refugees or increased military security measures in the armed conflict nearby. They sustained and supported, materially and morally, a large wave of refugees. In the future, the area’s population also estimates the need to align with additional security measures due to the military conflict near the border.

Following the “Reilly-Converse” model, it was deciphered that there are several centers of polarization and dominant flows in the analyzed zone. The pattern considers several criteria for ranking urban centers, such as the number of inhabitants, the position in the network of settlements, the range of services available in that center, the distance, etc. These criteria allow for observing the main spatial interaction movements at the regional level. The cities of Arad and Oradea have complex services of sub-regional importance, linked to four large and medium-sized cities, two from the North–West Region and the others from the West Region [1].

The current geo-socio-economic context favored the placement of the functional industrial activities in the analyzed region and has several strengths, such as the positioning on the country’s western border, easy access to Europe, and near the major development poles concerning the distribution of employed labor resources. Most of the employed people in the explored area worked and are working in the industrial sector [8].

We consider that active participation in the educational process of the adult population is essential for building a sustainable and competitive economy [9,10,11]. The argument for associating responsibility, especially with the activity rate of labor resources, is that accountability is one of the important qualities that an active person involved in daily professional and personal activities must possess. Tracking the total number of employees, the share of those working in industry (which represents more than 1/3 of the active workforce in the analyzed counties), their training level, and the activity rate of labor resources allow for appreciating the relevance of the liability analysis.

The present study began in the middle of the pandemic period, April–May 2021, when the region was under restrictions. All the limitations given by this context were assumed. Under these conditions, we applied the first online questionnaire through the Google Forms platform. The people who responded to our invitation to fill out the questionnaire were precious, responding in smaller numbers to our request. We believe that this was because they were, for the first time, under the pressure of restrictions, which they found more difficult to adapt to in the first stage of the pandemic. We resumed the research in April–May 2022. That was the period following the lifting of the constraints, but shortly after the beginning of the Ukraine conflict. Romania’s North–West area is not very far from the Ukrainian border. The questionnaire used in all these moments was the same, and the application method was online. The third part of the research took place in November–December 2022, bringing new elements regarding the ways of showing responsibility by the respondents in the functional area into the study. The research used the same instruments, but the sample was extended.

In this work, we set out to determine, from an empirical perspective, how responsibility is manifested as an element of assuming one’s actions and ethics towards peers and the environment in limited conditions imposed by external factors (wars and pandemics). This research describes the mechanism that triggers an increased responsibility of individuals by (1) determining the sensitivity of the responsibility of individuals to the action of an external factor (the COVID-19 pandemic at different times of evolution) and its intensity; (2) identifying the reasons for increasing responsibility; and (3) presenting some forms of manifestation of responsibility in the given socio-economic and environmental context.

The research unfolded over two consecutive years and was based on the following assumptions:

**Hypothesis 1 (H1)**: *The first one assumed that during the COVID-19 pandemic, the respondents became more responsible toward every aspect of life*.

The research confirmed this premise.

**Hypothesis (H2)**: *It was presumed that during the COVID-19 pandemic (research time 2021), the respondent became more committed, but only in certain aspects of life.*

The results from the 2021 investigation ascertained it.

The interviewees become more responsible through external constraints, considering certain situations such as critical/extreme ones and protecting health. The assumption determined the formulation of:

**Hypothesis 3 (H3)**: *External conditions have a lesser role in making people with a higher level of training responsible.*

The 2022 study results endorsed the initial presumption.

The study’s novelty comes precisely from high-impact social conditions determined by the sanitary situation, COVID-19, and the war positioned on Romania’s border, which European citizens have not faced in the last 60–70 years. The representative literature approaches similar topics, but it is important to consider the psychological constraints in such cases. The stress generated by the war becomes very important here.

## 2. Literature Review

**Figure 1:**
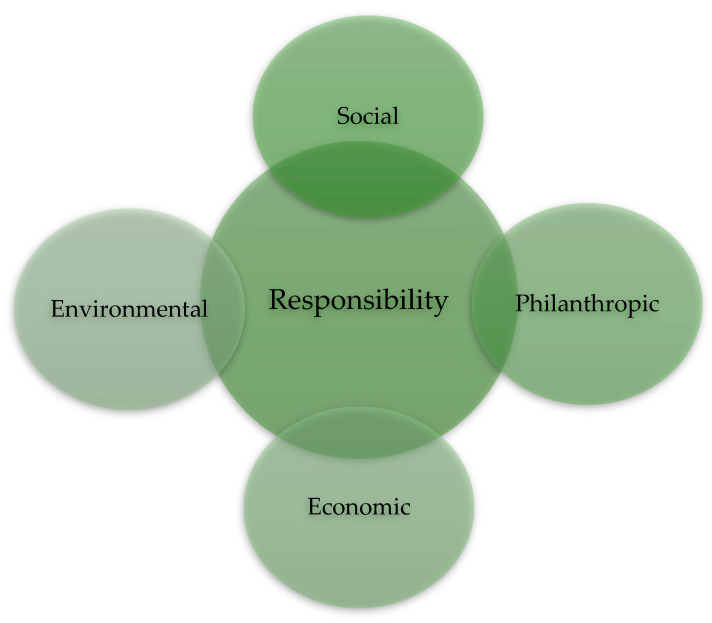
Forms of responsibilities.

The research for this article started precisely from two essential aspects. Firstly, accountability can be seen as an element of assuming exclusive personal actions. Secondly, it is considered an effect of ethics toward fellow citizens and the environment. These aspects have been used a lot since the beginning of the pandemic. For this reason, we wanted to observe the initiating processes that increase responsibility. It is important to determine if some instruments or systems determine liability or if it results from the educational and learning system. It was also interesting to differentiate the family principle’s effectiveness and the one achieved across the academic route. This aspect was considered from different points of view, as presented in Figure 1.

Etymologically, the word responsibility comes from both the French language “responsabilité” and the Italian language “responsabilitá”, being defined as a “conscious attitude, or sense of responsibility towards social obligations” [12]. In other words, being responsible means having a conscious attitude, assuming the actions you undertake, knowing how to explain why certain processes are performed, and accepting the consequences. Responsibility encompasses every area of our lives, going hand in hand with assuming our actions and making conscious and moral decisions. The present work does not refer only to the individual’s responsibility and ethics but also to social responsibility as an integral part of our activity.

Social responsibility means “the obligations of business people to follow those policies or directions, to make those decisions, that is agreed in terms of values and objectives by our society” [13].

Regarding the company’s social responsibility, it can be emphasized that it can take several forms depending on the activity’s specifics, the business model, the company culture, and the promoted values. In general, it is put into practice by involving them in activities that contribute to protecting the environment and improving the social and economic environment. All these aspects bring certain advantages to them, thus becoming better known on the market and having a better position in front of partners, authorities, or collaborators.

According to the European Community, it represents “the voluntary integration by companies of social and environmental concerns in their commercial operations and their relationships with interested parties” [14]. Practically, their main objective is to create sustainable relations with all the actors involved in the company’s activity, along with the economic responsibility [15], highlighting and promoting its values, the respect for the locals [16], the territory, and the environment. Each new business should include in its development plan different social responsibility elements [17]. The entry of social responsibility into the community will inevitably influence it [18]. Considering the recent events, all corporations had to adjust their internal view regarding the obligations towards their employees and society. The efforts guided them to implement durable actions for percussion situations [19].

Environmental responsibility is implemented through measures that reduce pollution or gas emissions, decrease and recycle waste, use resources sustainably or offset negative ecological effects through certain actions, such as reforestation. Javeed et al., in their study, imply the positive impact of such actions on the firm’s economic development [20]. The level of social implication is closely related to the company’s financial efficiency [21].

Those companies that voluntarily assume ethical responsibility consider that all interested parties are treated equally to share the same values, both managers and employees [22], as well as service providers or customers. Different studies, such as Aslan et al. or Nguyen et al., highlight the synergistic relationship between applying the corporate deontological principle and its social role [23,24].

The recent literature revealed some possible mechanisms contributing to citizens’ increased responsibility. Most of the efforts targeted the sanitary aspects. Importance was given to the communication channel used and the community relational scale. The desire to ensure the acquaintance’s well-being was higher in small groups than in large groups [25]. The exhibit of possible measures for a decrease in the level of contamination determined the voluntary application of protection measures to a higher degree.

Participants with a certain level of knowledge in the field of environmental protection easily accept the involvement in specific commonwealth projects [26]. Such a situation underlines the importance of the educational system in attitude formation. The considerable impact of higher education on youth perceptions regarding ethical behavior was also determined by Valente et al. in their study [27]. Rodríguez-Gómez et al. also point out the importance of transmitted family values [28].

Many companies adopt philanthropic responsibility, an extension of environmental and ethical responsibility, by donating goods for various purposes. An ascending economic trend creates a favorable environment for such an attitude [29]. Local perspectives could influence this attitude. A difficult family situation while growing up also affects an adult who becomes a successful manager. Based on parental education will decide his openness to adopting such attitudes [30].

Economic responsibility is the final goal of all activities carried out by companies. It is a regular part of their business plan. Thus, they focus on “profit, people, planet”. According to one of the definitions given by the European Union, it means “satisfying the demands of customers and at the same time knowing how to manage the expectations of other stakeholders: staff, suppliers and local targeted communities” [31].

This policy includes the opportunity to obtain benefits and advantages at the company level and in the context in which it conducts its activity, but also by developing partnerships with companies from the same sector or different sectors. It is necessary to emphasize the fact that there are two main dimensions of the company’s responsibility. An internal one includes managing human resources, health, and workplace safety. The external responsibility is based on interacting with the local community, suppliers, consumers, and economic partners. It ensures respect for human rights throughout the production chain and for environmental issues at the local and global levels.

Sustainability implies a change in process at the company’s level so that natural resources are used sustainably. Still, technological development and the exploitation of current and future potential are oriented in this responsible and durable direction, with full respect for the environment.

Different investigations assert that an eco-friendly view and approach determines personal transformation by enhancing responsibility to ensure a better future for the next generation [32]. From here comes the concern of adult individuals regarding the effects of the pandemic and the war on society and the environment, who pay greater attention to the future to correct waste management, care for air quality, etc.

When people assume responsibility for their actions, no matter what they are, they also undertake ethical behavior. Ethics is one of the main branches of philosophy. It deals with the research of moral issues, trying to answer questions such as what is good/bad. In the conception of individual values, ethics is synonymous with correctness, goodness, and good. Morality is often infringed on precisely because of the irresponsible and irrational acts of those around us.

Social responsibility has five principles on which it is based: transparency, quality, voluntarily adopted, integration, and sustainability. Every person who undertakes to implement these principles could be considered responsible and ethical. Therefore, being a responsible person is voluntary. He chooses to be transparent in his actions and integrates and respects the environment in which he carries out his activities and lives [33,34,35].

## 3. Materials and Methods

The research was carried out in three different instances between 2021 and 2022, having as its theme the individual responsibility shown by the active population, the employees in the industrial sector, from the North-West border area of Romania. The high activity rates of labor resources in the counties that surround this area and the significant share of employees working in the industry in the analyzed territory facilitated the formation of the sample of this sector. The active population in the functional industrial area of the four counties we are addressing is 182,397 employees [8].

In the first part of the research, conducted in April–May 2021, 173 respondents employed in the industry were interviewed, assuming all the risks and constraints of the situation generated by the COVID-19 pandemic existing at that time. Regarding the second stage of the research, in April–May 2022 and November–December 2022, the sample size was 409 respondents. At that time, sanitary restrictions were removed. The sampling procedure used in the two situations was non-random, non-probabilistic, with independent quotas [36]. The chosen stratification factors were the employees’ environment of origin, from the counties of Arad, Bihor, Maramureș, and Satu Mare, and their gender. The limitation imposed by the established variables facilitates the effectiveness of surveying in identifying the people who correspond to the indicated quotas [36], also increasing the analysis subjectivity. Knowing the distribution of the analyzed statistical population by gender and the environment of origin, the final selected sample had the same percentage distribution as the total population. The two stratification factors used led to the formation of independent quotas, in the sense that the division by gender is not linked to that by origin.

Knowing the distribution of the analyzed statistical population by gender and the environment of origin, the final selected sample had the same percentage distribution as the total population. The questionnaire distribution by county is as presented in Table 1.

We used primary data collection as a specific technique. The quantitative research method was applied to empirically validate the proposed hypotheses, with the questionnaire as the research tool. It was designed in such a way as to include questions classified according to content (factual, motivational, and opinion), but also queries classified according to their form: semi-open or closed, scaled. The questionnaire comprises six items. One item measured the sensitivity of the responsibility of individuals to the action of an external factor and its intensity using a five-point differential semantic scale. Five questions were of multiple-choice type, analyzing the other issues mentioned above of the mechanism that triggers an increased responsibility in individuals.

In addition to the questions regarding the research theme, we included inquiries related to variables such as age, gender, and level of studies. The survey was created online using the Google Forms platform. A standard protocol for administering the questionnaire was used [37].

The research was carried out on a non-probability sample with independent quotas. The stratification factors were chosen to be the employee’s environment of origin, urban–rural, from Arad, Bihor, Maramureș, and Satu Mare counties, as well as their gender. This approach helped us to achieve a diversity of respondents in terms of their level of education.

It could be observed that the percentage of the urban population is slightly higher, 51.43–56.89%, compared with the one from the rural part based on the statistics available for the counties included in the research. Urban centers such as Oradea, Arad, Satu Mare, or Baia Mare have expanded towards the metropolitan area, noticeable by an increase in population in peri-urban localities. The balance between the males and females tilts mildly toward the second one. Other aspects considered were the number of graduates. This approach helped us to obtain a diversity of respondents in terms of their level of education.

Based on these aspects, the data collection process was also organized using the Google Forms platform by inviting the type of respondents chosen according to the non-probability sample size based on the independent quotas established in the research and the employee’s background, urban or rural.

In the present study, we used the questionnaire to collect information because it captured the “social reality” when the research was conducted. In the period analyzed, 2020–2021, Romanian society was marked by the health crisis, the beginning of the Russia–Ukraine war, and individual and collective problems generated by the energy crisis. The questionnaire was administered using the Google Forms platform indirectly through self-administration. In this case, the interviewer did not influence the individual’s reactions. The respondent had time to think, recall events, and corroborate his own experiences with those close to him, thus favoring introspection on what was stated by the themes addressed and summarized as faithfully as possible what they thought it necessary to answer. Most of the time, these questionnaires were completed at home, i.e., where the respondent spends most of the time and where they lived the experiences recalled during the survey.

Other reasons why we decided to apply the questionnaire indirectly were related to the adaptation of the data collection process to the given situation, i.e., the COVID-19 pandemic situation and the restrictions imposed by it, as well as the diversity of issues addressed by the research topic, i.e., the territorial dispersion of respondents.

The reason for choosing non-probability sampling with independent quotas is primarily based on the fact that it facilitated the rapid application of the questionnaire (respondents are selected according to the indicated percentages), as there is no need to identify a specific respondent who needs to be interviewed correctly and be convinced to respond as in random sampling. The second reason for choosing this sampling was that no sufficiently well-developed sampling framework does not distort the population structure. Practical explanations of frequent use compensate for the lack of mathematical mechanisms to validate the sampling design.

After collecting the information, databases were created, and subsequent data analysis was performed in the SPSS program—IBM Statistics-Version 23, provided by IBM Corp. (Armonk, NY, USA). The Chi-Square Tests and *t*-tests used for independent samples were applied, allowing the effect size estimation. The *t*-test detects whether this research has statistical significance and is not a fluke. The expected error was ±5%, with a probability threshold of 95%. It assesses the differences between the observed values and those predicted if the population had no relationship. It is statistically sizeable and could be used to enhance correlations if there are [38].

We followed the scale recommended by Boateng et al. in their investigation to develop and validate health, social, and behavioral research [39]. First, we analyzed the existing theories regarding the initiation processes that increase responsibility in the individual and socio–economic–environmental context, differentiate the effectiveness of the family principle, and are carried out on the educational path. Then, we reviewed articles from the available literature to create six items corresponding to possible mechanisms that contribute to increasing the responsibility of citizens in vulnerable situations (the COVID-19 crisis). All articles have been validated; therefore, we sent our questionnaire to the respondents.

## 4. Results

As mentioned before, the research was carried out in three different periods. As a consequence, the respondents’ profiles are variable. So, around 44.00% of the respondents interviewed in 2021 were between 31 and 40 years old, and more than half were males. The majority (over 90%) have university and postgraduate education.

At the pandemic’s beginning of the pandemic, most of those who used online communication methods were those with higher education. Later on, this communication method was extended among the employed population and connected to the specifics of the work carried out.

In the second part of the research, from April to May 2022, the respondents’ age profile was approximately similar, with 44.00% between 31 and 40 years old and nearly 41.00% being male. The portion of the interviewees with university degrees decreased to about 71.00%.

For the last part of the research carried out in November–December 2022, 17.40% were between 31 and 40 years old, with females as the majority. The higher education graduates were close to the ones from April–May 2022, i.e., 67.20%.

The respondents’ profiles also reveal how the ability to use online communication methods evolved during the pandemic, comparing the respondents from the three research instances. In 2021, most of the respondents had higher education, a master’s or doctorate, fitting into the categories that normally used online communication methods all the time before the pandemic. The high school graduates that participated in the survey in 2022 were around a third compared to 2021, which had only 1.2%. It highlights that external constraints cause the respondents to improve their ability to use technology.

All graphical responses presented in the article were obtained from an open-ended survey. The questionnaire comprises six items. One item measured the sensitivity of the responsibility of individuals to the action of an external factor and its intensity using a five-point differential semantic scale. Five questions were of multiple-choice type, analyzing the other issues mentioned above of the mechanism that triggers an increase in responsibility in individuals.

**Figure 2:**
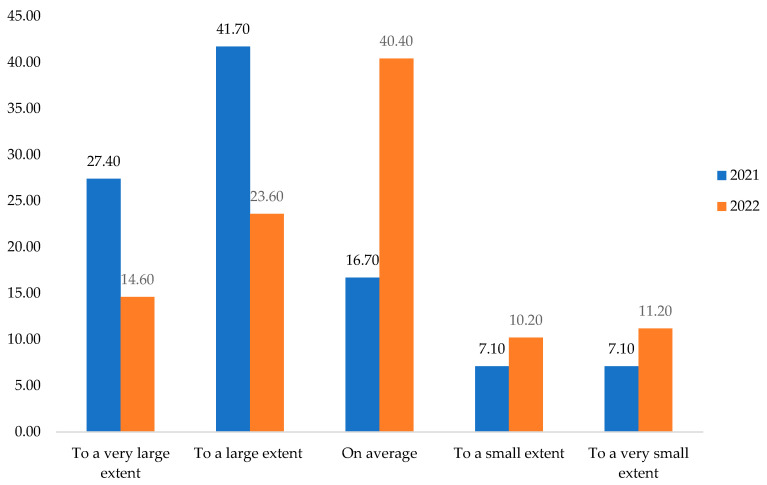
The degree of responsibility of North–West functional industrial area population during the COVID pandemic period. Source: Data from field quizzes, 2021 and 2022.

As previously shown, the first hypothesis (**H1**) claimed that the respondents became more responsible during the extended period of the COVID-19 pandemic. The assumption was confirmed through the research results. Thus, respondents were asked to what extent they believe they have become more responsible during the pandemic. It was found in 2021 that 27.40% thought they had become more reliable to a very large extent and 41.70% to a large extent. The percentages are higher compared to the statements from 2022. Also, in 2022, 40.40% considered that they were, on average, more responsible, while in 2021, only 16.70% thought that they were more accountable (Figure 2).

We note that following the application of Chi-Square Tests, the hypothesis was verified. Chi-Square Tests show that the obtained *p* < 0.005, Table 2, and the variables are associated, which means that the period of the COVID-19 pandemic influenced the respondents, making them more responsible.

The comparatively analyzed answers to the question about “the degree of responsibility of the population in the functional industrial area North–West of Romania” highlight that external events mostly lead to the increase in the degree of responsibility. These are very close in terms of temporality and spatiality to the respondents. As such, the more an external factor affects them directly, the more responsible they become. Thus, in Romania′s case, responsibility comes not from one’s development but from external compulsion. This aspect shows us the need for training and continuous personal and professional development at all social levels.

In general, all the elements related to the field in which the respondents more clearly manifested the responsibility in 2021 are completely different from the ones displayed in the latter year.

**Figure 3:**
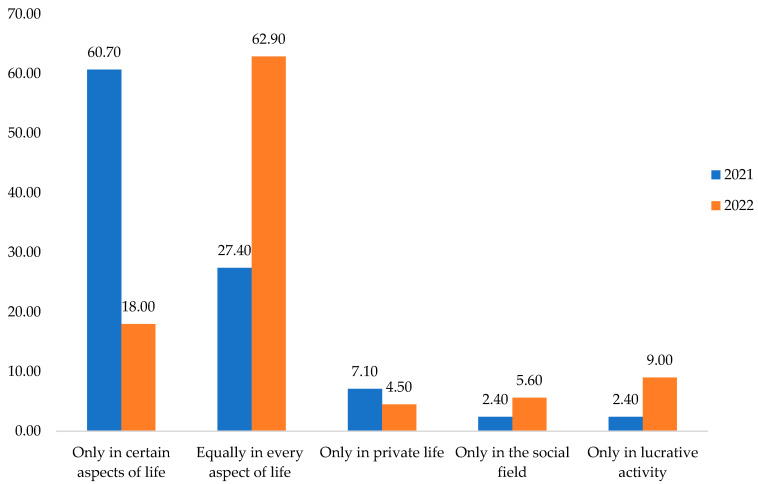
The responsibility manifestations of the population in the functional industrial area in the North–West of Romania during COVID-19 and post-COVID-19. Source: Data from field quizzes, 2021 and 2022.

Thus, from Figure 3, it can be seen that in 2021, only 2.40% gained more responsibility. In 2022, the proportion was 9%. In the social field, in 2021, 2.40% of respondents believed they were more responsible, and 5.60% in 2022. Regarding private life, 7.10% considered in 2021 that they were more accountable, and 4.50% in 2022. However, 27.40% in 2021 and 62.90% in 2022 were equally responsible in every moment of their lives. Responsibility was manifested only in certain aspects of life in proportion to 60.70% of respondents in 2021 and 18.00% in 2022.

In conclusion, hypothesis (**H2**), by which it was assumed that during the COVID-19 pandemic, the respondents became more responsible only in certain aspects of life, was confirmed by the research conducted in that year. Following the Chi-Square Test, Table 3, the variables are associated, and H2 is verified.

We could also observe that one year after the major external constraint, the COVID-19 pandemic, the respondents have become equally more responsible in every moment of their lives. We can say that the conscious attitude or the sense of responsibility towards social obligations and the actions people undertake has extended to all aspects of life. Thus, after an unfortunate situation, people learned that they need to be more aware of what they are doing with their lives, triggering the mechanism of self-defense and preservation.

These observations led us to extend the research to a period different from the COVID-19 pandemic. At the end of 2022, the respondents no longer vividly kept the pandemic period in their individual and collective memories. In addition, compared to the analyzed interval, November–December 2022, eight months have passed since Romania renounced the health restrictions and nine months since the beginning of the armed conflict on the country’s North–West and Northern borders. This situation coincided with some “habit” and “accommodation” of the population.

The manifestation of the pandemic and its implications in society highlights not only an increase in the degree of responsibility of the population but, to some extent, the perception of a certain post-COVID-19 vulnerability in certain social categories.

**Figure 4:**
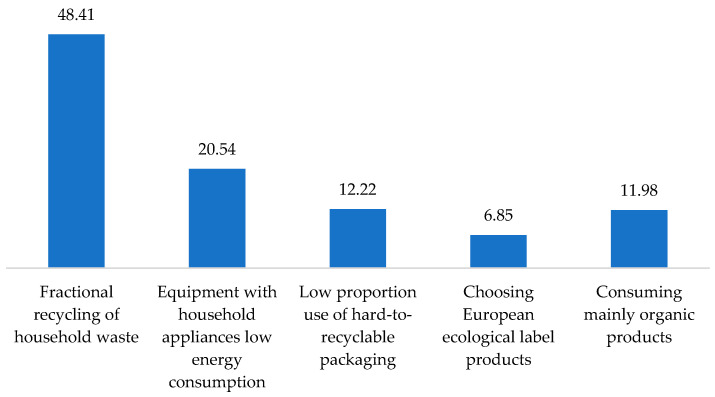
North–West Romanian functional industrial area’s population’s responsibility towards the environment in the post-COVID-19 period. Source: Data from field quizzes, 2022.

Considering these aspects, during the post-COVID period, we asked the respondents how they showed their responsibility towards the environment and through what kind of actions. Their answers were structured as follows: 48.41% differentiated household waste during recycling, 20.54% equipped their households with appliances that had low energy consumption, 12.22% used packaging that is easier to recycle, 12.22% preferred to consume mainly biological products, and 6.85% chose products with a European ecological label (Figure 4).

**Figure 5:**
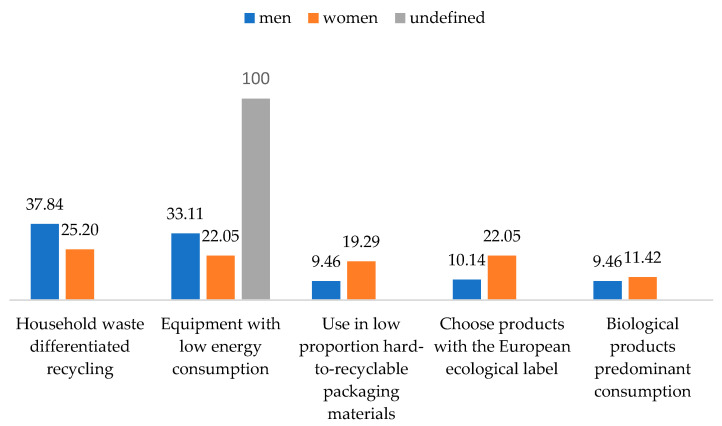
Eco-friendly actions that the respondents performed to decrease their personal environmental impact. Source: Data from field quizzes, 2022.

Correlating the way to be more responsible towards the environment with the gender of the respondents allowed us to observe that both males (37.84%) and females (25.20%) chose to differentiate recycling of household waste. Approximately a third (33.11%) of men equipped their household with appliances with low energy consumption. Around one-fifth (22.05%) of women preferred consuming products with a European ecological label and organic products (Figure 5).

**Figure 6:**
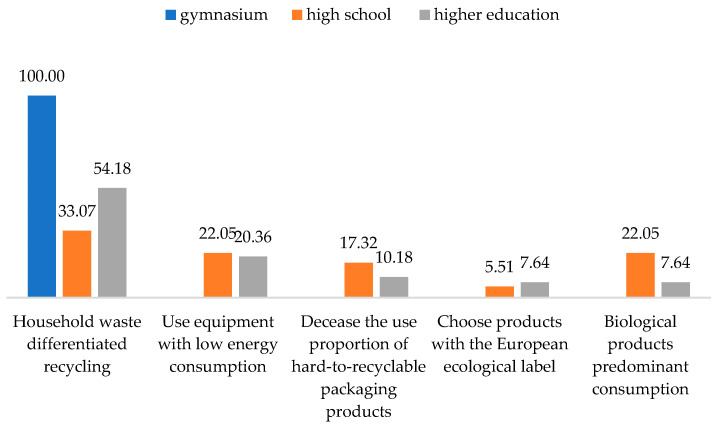
Correlation between the eco-friendly actions that the respondents performed to decrease their environmental impact and their education level. Source: Data from field quizzes, 2022.

Subsequently, we correlated the ways followed to become more responsible towards the environment and the level of training. It was observed that as the level of education increased, the forms of action regarding responsibility towards the environment also diversified (Figure 6). Thus, the respondents chose to have a liable behavior towards the environment and be sustainable simultaneously. The attitude manifested through differentiation of household waste for recycling was identified in all those with a secondary school degree and only in half with higher education. Another important segment of the respondents chose household appliances with low energy consumption. Among those, 22.05% had a high school diploma, and almost the same percentage had higher education. From the last category, only 10.18% were interested in decreasing the use of packaging and hard-to-recycle products.

Responsibility is closely related to sustainability and education. The study shows a direct correlation between the instruction level and durable actions.

Correlating the respondents’ gender with the reasons that led them to be more responsible led us to observe that the main reason our irresponsible actions determine the negative effects on the environment was agreed on by over half of the males and only one-fifth of women. Then, for women, a proportion of 38.58% believed that concepts they learned from their family at an early age helped them improve their responsibility over time. Instead, only 18.92% of male respondents agreed with this statement.

Given the data presented and the literature inputs, sustainable development aims to ensure the survival and quality of life for all species, and here we are not talking only about humans, so it became a valuable instrument that could balance the economic, social, and environmental components. Considering a global perspective, beginning with the first law of ecology issued by Commoner, ”Everything is connected to everything else”, where under the same roof of the ecosphere, all living organisms are interconnected, and what affects one will affect all of them; furthermore [40], continuing with Gaia theory of Lovelock, where all living organisms of ecosphere interact with their surrounding inorganic environment, resulting in a synergistic and automated system, it is clear that a moral and ethical perspective needs to be adopted [41]. We need to answer major questions for each sustainable development aspect, like who is making decisions and who is consulted. To what extent, who are the main beneficiaries, and who is going to lose? And here again, we are not taking into account only human civilization, under what values we will govern and whose values, which aspect of our life will gain benefits, what quality of life should look like, which risks are we taking and especially under global crisis like a pandemic, what matters more, what is a priority.

An ethical component will assure fairness to all humans, present and future generations, and all environmental spheres in which we coexist. The studies of Busoi et al. emphasize that sustainable development without morality cannot exist due to its major implications on social life [42].

**Figure 7:**
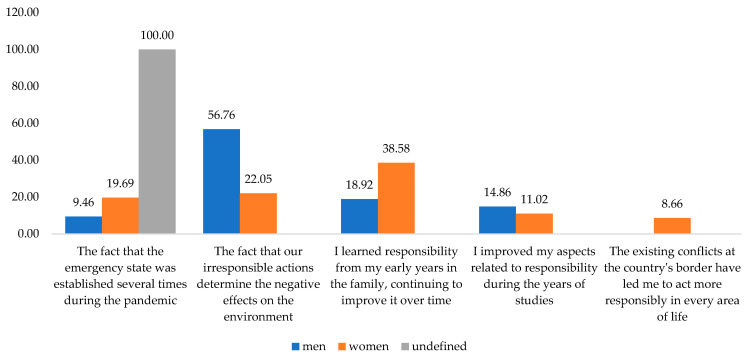
Reasons that led the respondents to become more responsible, given their gender. Source: Data from field quizzes, 2022.

The external constraints given by the existing conflicts on Romania’s border caused only 8.66% of the female respondents to be more accountable during 2022 (Figure 7).

The restrictions introduced due to COVID-19 pandemic are still impact responsibility. The proportion is almost 20% among females and 10% among males.

**Figure 8:**
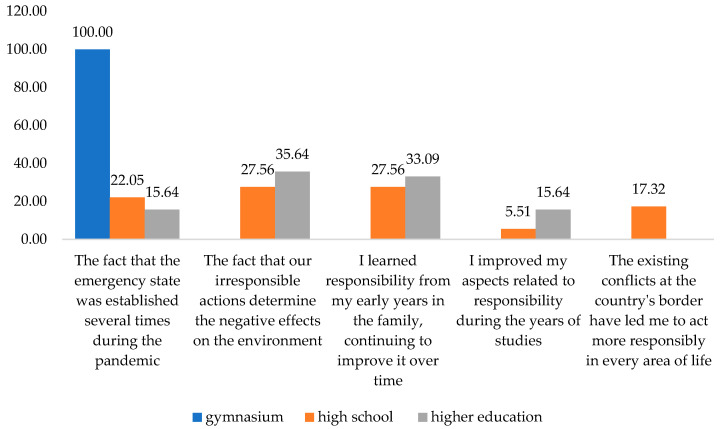
Firstly, considering the respondents’ education level, the reasons that led them to become more responsible. Source: Data from field quizzes, 2022.

Another correlation established in this paper was between the reasons that led the respondents to be more responsible and their level of training. The association confirmed the third hypothesis of the research. It was assumed that external constraints have a lower role in the responsibility of people with a higher level of training (**H3**). As shown in Figure 8, the fact that the state of emergency was established several times during the pandemic was chosen by 22.05% with a high school education level as their first response. Among the ones with higher education, the percentage was lower (15.64%). As a second reason, the mentioned answer was chosen by 6.31% of the first-category respondents and 10.54% of the second-category respondents, respectively.

As the first choice, the answer was chosen by 27.56% of those with a high school diploma. The one with higher education represented a percentage of 35.64%. One of the reasons that led the interviewees to be more responsible is I learned responsibility from my early years in the family, continuing to improve it over time.

The data analyzed provide a convincing argument on the implications of responsibility in times of crisis, especially through the lens of Romanian society. The external conditions considered to influence the people’s empowerment were provided in the socio-economic and environmental context in the research period 2021–2022. In this regard, we thought about several critical circumstances, such as the health restrictions imposed by the COVID-19 pandemic (a state of emergency that brought with it isolation at home and the imposition of work from home), the armed conflict between Russia–Ukraine, the Romanian border, and the danger of its spread (which also generated the feeling of insecurity, fear about the future), and also environmental problems (pollution and global warming) concerning the energy crisis.

The research confirms that studies play an active role in forming and developing individual responsibility. Analyzing the impact of the level of training on commitment, we also notice that the respondents with a high school education in a proportion of 5.51% and 15.64% of those with a university education support the statement that they improved on aspects related to responsibility during the years of study. The second answer option is the option selected by 27.56% of those with high school education and 28.36% of higher education graduates.

**Figure 9:**
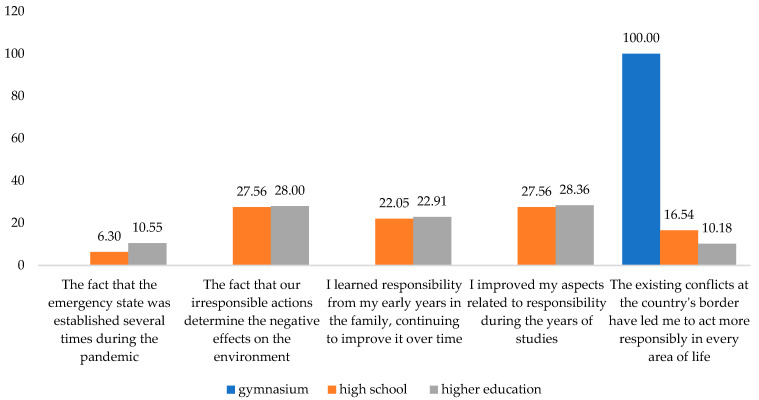
Secondly, considering the respondents’ education level, the reasons that led them to become more responsible. Source: Data from field quizzes, 2022.

The constraint represented by the existing conflicts at the border of the country has made me determined to act more responsibly in every area of life was chosen as the first answer by only 17.32% of the respondents with a high school diploma. All the gymnasium graduates have chosen as second motivation the same alternative. Compared with them, the situation was different for the high school and higher education graduates, 16.54% and 10.18%, respectively (Figure 9). Thus, the third hypothesis is confirmed.

Several important aspects are revealed by analyzing these data. The first premises for becoming a responsible adult are initiated in the first years of life in the family. Where there has been increased attention on education, it has continued throughout life. On the other hand, once accountability is instilled, it continues to widen its applicability as the horizon of knowledge expands. Higher education adds value to a person’s responsibility attribute [43]. It opens the possibility for educational and cultural experiences through transnational activities, scholarships, training, and offered project opportunities [44].

## 5. Discussion

The issue of responsibility appears as a major element in the foundation of the sustainable course of humanity. Accountability, in general, is one of the important qualities that must be possessed by a person involved in daily professional and personal, individual, and/or collective activities in close relationship with the environment and society. For this reason, the analysis and knowledge of its triggering mechanism and the endogenous and exogenous factors that influence the individual and the collective remain topical.

The last year’s retrospection was dominated by successive crises that society has gone through and is still going through. In general and in the Romanian community in particular (irrespective of the health crisis-the COVID-19 pandemic from 2020–2022, the current energy crisis, and the armed conflict on the border of Romania), we were able to carry out this study at the level of the active population in the border area of North–West Romania (counties of Arad, Bihor, Satu Mare, and Maramureș). The research results of inhabitants located in areas sensitive to external constraints, i.e., the pandemic crisis and the armed conflict of Russia–Ukraine situated in their vicinity, highlighted more of a mechanism of the formation of responsibility that people feel in borderline situations. It is presented as an alternative to tools useful to transform such circumstances into a sustainable and effective commitment (resilience) through formal and non-formal lifelong education.

All the new constraints emphasize the need to find short-, medium--, and long-term solutions for specific segments (health, energy, environmental quality, defense, security, etc.). As a rule, short-term measures have an unfavorable impact on the population from a psychological point of view. Still, they realize that medium- and long-term measures support or favor them.

The research opens new study perspectives for this subject that can be extended to the European cross-border areas. For example, the Danube–Mureș–Criș–Tisa cross-border area can be included in studies related to the implications of responsibility as an element of ethics and sustainability of the European space and even in the situations facing humanity linked to the ethnic coexistence of different people, cultures, and minorities.

## 6. Conclusions

Following the research carried out, the hypotheses were confirmed. Thus, it can be stated that this study clarifies how to transform people’s responsibility in certain borderline situations into a sustainable and effective commitment independent of such conditions and unfavorable external factors. This renewal can only occur through training, continuous personal and professional development, and awareness of the skills and competencies. These contribute to having a conscious attitude and assuming actions, knowing how to explain the reasons for which activities were undertaken while accepting their consequences.

Conscious formal and non-formal learning also represents a generating source of value and knowledge, which, appropriated and then applied, can generate new business models oriented more towards the development of eco-technologies and can revitalize companies in which people, the relationships between them and the social responsibility focus on the learning approach.

The study also shows that the sense of responsibility towards social obligations and the actions that people undertake has extended to all aspects of life following the intervention of exogenous factors in the last three years. This fact can trigger the greater assumption of ethics in business, both in the private sector and in the administration, and, by extension, in creating a framework for the manifestation of philanthropic responsibility.

The T-test results also support that the respondents were more responsible at the time of the exacerbation of the pandemic (2021) and less responsible when the restrictions were lifted (2022). But, conscious learning from experience generated by external constraints is not enough. Deliberate, continuous, planned, and organized learning/training is necessary for formal and non-formal environments for all individuals precisely to support sustainable development and the existence of an “ethical” society.

It is necessary to change mentalities in the future, based on the acquisition of the “lessons” received by society (health problems, economic crises, natural disasters, armed conflicts, environmental issues, etc.) and also to improve the mechanisms of consciousness, responsible action at the level of the entire society, regardless of the given situations.

Conscious formal and non-formal learning also represents a generating source of value and knowledge. Their appropriate application can generate new business models—oriented more towards developing eco-technologies and revitalizing companies in which people, the relationships between them, and the social responsibility focus on the learning approach.

The study also shows that the sense of responsibility towards social obligations and the actions people undertake has extended to all aspects of life following the intervention of exogenous factors in recent years. This fact can trigger the greater assumption of ethics in business and, by extension, in the framework creation of a manifestation of philanthropic responsibility.

Also, from the paper, we conclude that economic responsibility, treated from a worldly perspective of profit, is redefined in the context of the action of exogenous factors (health crisis, armed conflicts, global warming, and energy crisis). It transforms from an individualistic approach to a collaborative one, oriented more towards staff, suppliers, local targeted communities, and even competitors, in general, towards the people’s soul and spirit.

All these aspects, presented in the work, make individuals and society as a whole determined to pay more attention to future problems that may arise and past and present lessons to help make faster and more correct decisions, both on a personal level and on the community level that we are part of.

Responsibility is part of soft abilities, together with flexibility, adaptability, respect, communication, etc. Some of this paper’s authors were part of the international team of the ERASMUS+ STRATAGAME Project (https://stratagame.erasmus.site/ro/ (accessed on 20 June 2023)), a Strategic Partnership For Soft Skills Building Through Gamification. It is considered that responsibility is a skill that can be learned through such projects and/or continuous training. Through education, a society can implement all-important changes needed to achieve the targeted collective proactive attitude [45].

The STRATAGAME project’s main objectives were to create effective and innovative tools that support all those who want to develop competencies in transversal and soft skills. They can be used by teachers for professional training and by those in continuing education programs. Two didactic digital tools have been created, i.e., the diagnostic test that assesses soft skills and the soft skills game, and can become a common methodology for introducing those skills in teaching programs. These are intended to build a common, systematic approach to initial and continuing professional development (CPD) for VET teachers by developing a training tool to expand users‘ soft skills through digital guides.

The skills addressed through this project are adaptability/flexibility, teamwork, creative thinking, problem-solving, and communication, and each of these categories is developed through other skills such as empathy, active listening, leadership, respect, commitment, decision-making, working with others, assertiveness, levels at which communication takes place, types of statements, elements that cancel it out, and connection pathways such as language and connecting in a creative relationship are just some of the aspects addressed.

We believe that such interactive, unconventional ways of education can be an alternative for those who want to be responsible and act ethically towards their fellow human beings and the environment.

A limitation of this research is represented by the fact that only one item was used to measure the sensitivity of the responsibility of individuals to the action of an external factor. The complexity of this construct may not have been fully evaluated.

Another limitation of the research is related to the small number of quotas (only two) used in the non-random sampling of the analyzed statistical population. Using a larger number of stratification factors could restrict the subjectivity of the research procedure. Too many factors could constitute an obstacle in research.

Methodological improvements to the research can be made under normal research conditions. It should be borne in mind that for a large part of the study period, April–May 2021 and April–May 2022, we faced several restrictions imposed by the COVID-19 pandemic, which also brought about the limitations indicated in the study.

The research could be expanded by using probability sampling, which would make the study more objective, diversifying the survey technique questionnaire, which can be developed under normal conditions with one applied face-to-face, but also with individual and group interviews, which would allow an in-depth analysis of the sensitivity of the respondent’s responsibility towards endogenous factors and their collective responsibility.

The analysis of how responsibility is evolving as an element of ethics and sustainability in Romania and beyond is our current and future concern.

In the case of Romania, perhaps more than in other European countries, action must be taken in the direction of cooperation between corporate and individual responsibility because responsibility began to manifest itself in the last 15–20 years [46].

Thus, through its models and actions, corporate responsibility can cause individuals to act in a certain way to guide them toward a certain behavior (through social activities, volunteering, involvement in public administration issues, etc.). On the other hand, individual responsibility (developed through education and an individual’s choices between good/bad and ethical or not) can determine the dimension of duty at the societal level.

In the case of Romania, we consider it important to promote:

- actions related to responsibility in all its forms;
- the concepts of sustainability and sustainable development;
- educational programs to help future generations based on these principles.

## Figures and Tables

**Table 1:** The survey form distribution covers the considered counties.

Total Survey No. by Year		Arad		Bihor		Maramureș		Satu Mare	
		M	F	M	F	M	F	M	F
2021	173	25	25	28	28	21	19	15	12
2020	409	58	55	66	64	53	48	36	29

M—male; F—female.

**Table 2:** Chi-Square Test for the question: To what extent do you think you have become more responsible during the pandemic?

	Value	df	Asymptotic Significance (Two-Sided)
Pearson Chi-Square	17.428 ^a^	4	0.002
Likelihood Ratio	17.842	4	0.001
Linear-by-Linear Association	9.251	1	0.002

^a^ Zero cells (0.0%) have an expected count of less than five. The minimum desired count is 7.28.

**Table 3:** Chi-Square Tests for Consider that this responsibility has manifested itself more clearly, in which area/aspect of your life.

	Value	df	Asymptotic Significance (Two-Sided)
Pearson Chi-Square	37.241 ^a^	4	0.000
Likelihood Ratio	38.880	4	0.000
Linear-by-Linear Association	16.883	1	0.000

^a^ Four cells (40.0%) have an expected count of less than five. The minimum expected count is 3.40.

## Data Availability

The authors will make the raw data supporting the conclusions of this study available without restriction.

## References

[1] Agenţia de Dezvoltare Regională (ADR) Nord-Vest Planul de Dezvoltare Regională Nord-Vest 2021–2027.

[2] EUR-Lex (2003). Nomenclature of Territorial Units for Statistics. Off. J. Eur. Union.

[3] O’Connor C., O’Connell N., Burke E., Dempster M., Graham C.D., Scally G., Zgaga L., Nolan A., Nicolson G., Mather L. (2021). Bordering on crisis: A qualitative analysis of focus group, social media, and news media perspectives on the Republic of Ireland-Northern Ireland border during the ‘first wave’ of the COVID-19 pandemic. Soc. Sci. Med..

[4] Oksanen A., Oksa R., Savela N., Mantere E., Savolainen I., Kaakinen M. (2021). COVID-19 crisis and digital stressors at work: A longitudinal study on the Finnish working population. Comput. Hum. Behav..

[5] Osiichuk M., Shepotylo O. (2020). Conflict and well-being of civilians: The case of the Russian-Ukrainian hybrid war. Econ. Syst..

[6] Alix-Garcia J., Schechter L., Valencia Caicedo F., Jessica Zhu S. (2022). Country of Women? Repercussions of the Triple Alliance War in Paraguay. J. Econ. Behav. Organ..

[7] Buchner J., Butsic V., Yin H., Kuemmerle T., Baumann M., Zazanashvili N., Stapp J., Radeloff V.C. (2022). Localized versus wide-ranging effects of the post-Soviet wars in the Caucasus on agricultural abandonment. Glob. Environ. Chang..

[8] National Institute of Statistics Statistics—FOM105F. http://statistici.insse.ro:8077/tempo-online/#/pages/tables/insse-table.

[9] Rees G., Peterson P., Baker E., McGaw B. (2010). The Political Economy of Adult Education. International Encyclopedia of Education.

[10] Klapper R.G., Fayolle A. (2023). A transformational learning framework for sustainable entrepreneurship education: The power of Paulo Freire’s educational model. Int. J. Manag. Educ..

[11] Brissett N.O.M., Tierney R.J., Rizvi F., Ercikan K. (2023). The education Sustainable Development Goal 4: A critical appraisal. International Encyclopedia of Education.

[12] Dexonline. https://dexonline.ro/.

[13] Bowen H. (1953). Toward Social Responsibilities of the Businessman.

[14] FourStars Impresa Sociale Responsabilità Sociale d’Impresa: Cos’è, Come Nasce, Quali Sono i Valori. https://www.4stars.it/blog/responsabilita-sociale-dimpresa.

[15] Sayekti Y. (2015). Strategic Corporate Social Responsibility (CSR), Company Financial Performance, and Earning Response Coefficient: Empirical Evidence On Indonesian Listed Companies. Procedia-Soc. Behav. Sci..

[16] Guirado C., Valldeperas N., Tulla A.F., Sendra L., Badia A., Evard C., Cebollada À., Espluga J., Pallarès I., Vera A. (2017). Social farming in Catalonia: Rural local development, employment opportunities and empowerment for people at risk of social exclusion. J. Rural Stud..

[17] Sergeeva N., Kapetanaki E. (2022). Corporate social responsibility as a strategic narrative: The cases of UK project-based organisations. Proj. Leadersh. Soc..

[18] Chen C., Wang L., Zhang Y. (2023). Do short-lived companies need to consider a social license to operate? Learning from an urban renewal project in China. Sustain. Prod. Consum..

[19] Forcadell F.J., Aracil E. (2021). A purpose-action framework for Corporate Social Responsibility in times of shock. J. Clean. Prod..

[20] Javeed S.A., Latief R., Cai X., San Ong T., Qian S., Haq A.U. (2022). What is the role of the board sustainable committee for corporate social responsibility? The moderating effect of gender diversity and ownership concentration. J. Clean. Prod..

[21] Saridakis C., Angelidou S., Woodside A.G. (2023). How historical and social aspirations reshape the relationship between corporate financial performance and corporate social responsibility. J. Bus. Res..

[22] Baker T.L., Hunt T.G., Andrews M.C. (2006). Promoting ethical behavior and organizational citizenship behaviors: The influence of corporate ethical values. J. Bus. Res..

[23] Aslan Ş., Şendoğdu A. (2012). The Mediating Role of Corporate Social Responsibility in Ethical Leader’s Effect on Corporate Ethical Values and Behavior. Procedia-Soc. Behav. Sci..

[24] Nguyen N.T.T., Nguyen N.P., Thanh Hoai T. (2021). Ethical leadership, corporate social responsibility, firm reputation, and firm performance: A serial mediation model. Heliyon.

[25] Hao F., Shao W., Huang W. (2021). Understanding the influence of contextual factors and individual social capital on American public mask wearing in response to COVID–19. Health Place.

[26] Sattayapanich T., Janmaimool P., Chontanawat J. (2022). Factors Affecting Community Participation in Environmental Corporate Social Responsibility Projects: Evidence from Mangrove Forest Management Project. J. Open Innov. Technol. Mark. Complex..

[27] Valente M., Sá C., Soares N., Sousa S. (2021). Exploring the consistency of ethical perceptions by business and economics higher education students: Looking from academia towards the corporate world. Int. J. Manag. Educ..

[28] Rodríguez-Gómez S., López-Pérez M.V., Garde-Sánchez R., Arco-Castro L. (2022). Increasing the commitment of students toward corporate social responsibility through higher education instruction. Int. J. Manag. Educ..

[29] Zhou C. (2022). Global diversification, host-country environments, and corporate philanthropic giving: Evidence from Chinese multinational corporations. Technol. Forecast. Soc. Chang..

[30] Han Y., Chi W., Zhou J. (2022). Prosocial imprint: CEO childhood famine experience and corporate philanthropic donation. J. Bus. Res..

[31] Award S. Che Cosa è la Responsabilità Sociale. https://sustainabilityaward.it/che-cosa-e-la-responsabilita-sociale-dimpresa/.

[32] Cuc L.D., Pelau C., Szentesi S.-G., Sanda G. (2022). The impact of green marketing on the consumers’intention to buy green products in the context of the green deal. Amfiteatru Econ..

[33] Swaner L.E. (2005). Educating for Personal and Social Responsibility: A Review of the Literature. Lib. Educ..

[34] Wikler D. (2002). Personal and social responsibility for health. Ethics Int. Aff..

[35] López Davis S., Marín Rives L., Ruiz de Maya S. (2017). Introducing Personal Social Responsibility as a key element to upgrade CSR. Span. J. Mark.-ESIC.

[36] Rotariu T., Iluț P. (1997). Ancheta Sociologică și Sondajul de Opinie. Teorie și Practică.

[37] Schleyer T.K., Forrest J.L. (2000). Methods for the design and administration of web-based surveys. J. Am. Med. Inform. Assoc..

[38] McHugh M.L. (2013). The chi-square test of independence. Biochem. Med..

[39] Boateng G.O., Neilands T.B., Frongillo E.A., Melgar-Quiñonez H.R., Young S.L. (2018). Best practices for developing and validating scales for health, social, and behavioral research: A primer. Front. Public Health.

[40] Commoner B. (2020). The Closing Circle: Nature, Man, and Technology.

[41] Lovelock J. (2000). The Ages of Gaia: A Biography of our Living Earth.

[42] Busoi S.M. (2015). Sustainable development and the influence of social values A case study on Romania. Procedia Econ. Financ..

[43] Berei E.B. (2020). The social responsibility among higher education students. Educ. Sci..

[44] Jones E., Leask B., Brandenburg U., de Wit H. (2021). Global social responsibility and the internationalisation of higher education for society. J. Stud. Int. Educ..

[45] Lazariuc C. (2022). Relația mentalitate colectivă–responsabilitate socială în condițiile noilor provocări societale. Cercetarea, Inovarea și Dezvoltarea din Perspectiva Eticii Globale.

[46] Veringă G., Veringă P. (2019). Responsabilitate socială corporativă și dezvoltare durabilă cazul României. Ecostudent-Rev. Cercet. Ştiinţifică Studenţilor Econ..

